# Obstetrics for Nurses

**Published:** 1904-08

**Authors:** 


Obstetrics for Nurses. By Joseph B. De Lee, Professor of Obstetrics in the Northwestern University Medical School, Chicago. Duodecimo, fully illustrated. Philadelphia, New York and London: W. S. Saunders & Company. 1904. (Cloth, $2.50 net.)

This book is written primarily for nurses, but the author says in his preface, and the reviewer heartily agrees with him, that medical students will find something of value in it. It is well written,—clear, concise, full. The illustrations are mainly original and from photographs. The book is to be commended to instructors of nurses as a textbook to be studied in conjunction with the didactic and clinical teaching of obstetrics. M. J. F.

